# Homogeneous Nuclear Background for Mitochondrial Cline in Northern Range of *Notochthamalus scabrosus*

**DOI:** 10.1534/g3.113.008383

**Published:** 2013-12-17

**Authors:** Christina Zakas, Ken Jones, John P. Wares

**Affiliations:** * New York University, Center for Genomics and Systems Biology, New York, New York 10003; † University of Colorado, Aurora, Colorado 80045; ‡ University of Georgia, Athens, Georgia 30602

**Keywords:** RAD-seq, SNP, cytonuclear disequilibrium, barnacle, Chile

## Abstract

A mitochondrial cline along the Chilean coast in the barnacle *Notochthamalus scabrosus* suggests a species history of transient allopatry and secondary contact. However, previous studies of nuclear sequence divergence suggested population genetic homogeneity across northern and central Chile. Here, we collect single-nucleotide polymorphism data from pooled population samples sequenced with restriction site−associated DNA sequencing procedures, confirm these data with the use of a GoldenGate array, and identify a discordance between population genetic patterns in the nuclear and mitochondrial genomes. This discordance was noted in previous work on this species, but here it is confirmed that the nuclear genome exhibits only slight phylogeographic variation across 3000 km of coastline, in the presence of a strong and statistically significant mitochondrial cline. There are nevertheless markers (approximately 5% of nuclear single-nucleotide polymorphisms) exhibiting cytonuclear disequilibrium relative to mitotype. Although these data confirm our previous explorations of this species, it is likely that some of the nuclear genomic diversity of this species has yet to be explored, as comparison with other barnacle phylogeography studies suggest that a divergence of similar magnitude should be found in the nuclear genome somewhere else in the species range.

Patterns of mitochondrial diversity have heralded great discoveries in biodiversity and often are used to indicate the likelihood that evolutionarily distinct lineages are harbored within recognized morphological species (Hebert *et al.* 2004; Moritz and Cicero 2004). However, divergence of mitochondrial lineages is not necessarily representative of the rest of a eukaryotic genome (Hudson and Coyne 2002; Hickerson *et al.* 2006). In some cases, exploration of divergence at nuclear markers supports a general pattern (Tellier *et al.* 2009), but in other cases the two genomic compartments appear to have wildly distinct histories of isolation and/or selection (Toews and Brelsford 2012; Pavlova *et al.* 2013). For example, divergence of mitochondrial lineages in an Australian robin (*Eopsaltria australis*) exhibits an east-west pattern, perpendicular to the latitudinal structure of nuclear markers (Pavlova *et al.* 2013).

Another such case of discordance has been identified in the barnacle *Notochthamalus scabrosus* (Foster and Newman 1987) along the central coast of Chile (Zakas *et al.* 2009). Two divergent mitochondrial lineages are found in this species, with one of them only found in natural populations south of ~30°S (Zakas *et al.* 2009; Laughlin *et al.* 2012). A nuclear gene (elongation factor 1-α; nEF1) surveyed for the same region showed no such geographic pattern of genetic diversification (Zakas *et al.* 2009), despite the capacity for this gene region to mirror such patterns and magnitudes of divergence in other coastal barnacles (Sotka *et al.* 2004). Because the nEF1 data did not exhibit the expected genealogical divergence, as seen in other barnacle species with similar mitochondrial clines (Sotka *et al.* 2004), it is clear that a better representation of diversity and divergence in the nuclear genome will be needed to understand the phylogeography of *N. scabrosus*.

Such a result suggests a discordant history for the mitochondrial and nuclear genomes of *N. scabrosus*, but of course one nuclear locus is not sufficiently representative of the dynamics of the genome. Recent advances in DNA sequencing technology (Schuster 2008) make it readily possible to efficiently survey a larger fraction of the genome for population genetic analysis. Elucidating the pattern of population divergence by the use of many thousands of nuclear loci reveals the potential combinations of ecological and evolutionary forces that drive this mismatch in genome histories. Here we use a combination of reduced-representation genome sequencing (restriction site−associated DNA sequencing, or RADseq; Peterson *et al.* 2012) of population samples and direct multilocus genotyping of markers developed from such data to find nuclear regions that may be associated with the divergent mitochondrial lineages found in *N. scabrosus*. Because the aforementioned nEF1 data did not exhibit a cline, we were interested in determining the extent to which a large representation of the nuclear genome displayed a concordant pattern. We also assess the utility of pooled RADseq data by comparison with direct genotyping of individuals in a subset of populations; the opportunity to collect appropriate data via pooled sampling, if it provides results consistent with individual genotypes, could be an efficient and inexpensive way to identify genome-wide diversity in nonmodel organisms.

## Methods

### Specimen collection and sequencing

**Figure 1:**
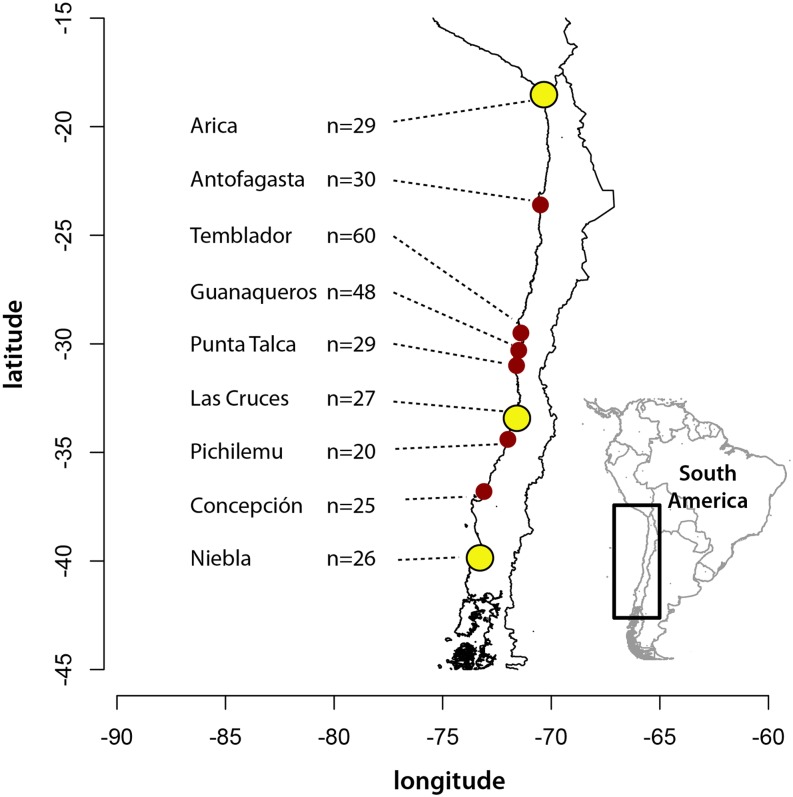
Locations of *Notochthamalus scabrosus* collections used in RADseq and SNP analysis (sample sizes indicate input for one or more libraries from each location for RADseq). All locations were included in RADseq analysis; sites marked in yellow were used for GoldenGate SNP analysis.

Collected specimens and DNA isolation protocols are described in detail in Laughlin *et al.* (2012). Sample locations are shown in Figure 1. Pooled samples of each location, with separate pools for “low” and “high” intertidal samples from Temblador and Guanaqueros, were generated for each site with equimolar concentrations of each individual. All individuals included had been previously sequenced at mitochondrial cytochrome oxidase I (COI) and identified to mitochondrial type. Three separate genomic DNA pools were generated for individuals comprised solely of the A1, A2 (“northern”), and B (“southern”) mitochondrial haplotypes as described in Laughlin *et al.* (2012).

Pooled genomic DNA were cut per standard protocols (Peterson *et al.* 2012) by the use of *SpeI* and *Sau3AI*, chosen for their utility in similar projects (K. Jones, unpublished results). Libraries were then prepared for Illumina sequencing by the use of TruSeq adapters and 100-bp paired ends were sequenced on an Illumina HiSequation 2500 at the University of Colorado at Denver core facility.

### Genome-wide single-nucleotide polymorphism (SNP) analysis

RADseq data were trimmed to remove adapter sequence and error-prone regions. Sequence fragments were aligned and organized with the program STACKS (Catchen *et al.* 2013). Because these data represent pooled samples from distinct locations, the consensus stacks were used as the reference library in the program Popoolation2 (Kofler *et al.* 2011), which aligns reads from pooled libraries to these reference sequences and generates allele frequency and other population genetic statistics such as F_ST_. SNPs were only included for analysis with a minimum fragment coverage of 50 and minimum quality score of 20.

### SNP selection

To genotype at the individual level, SNPs were chosen for assay using the Illumina BeadXPress platform as in Zakas and Wares (2012). Thus, SNPs were identified that had sequenced flanking regions of >30 bp for primer design; these were scored by Illumina for their probability of success. Only SNPs with the greatest possible quality ranking were selected for further analysis. We chose 192 for multilocus genotyping, setting specific *a priori* criteria for selection following Zakas *et al.* (2012), where ribosomal markers were eliminated and a balance of SNPs from coding regions and anonymous regions, as well as apparent nonsynonymous polymorphisms and synonymous polymorphisms were chosen. Consensus sequences for each region were evaluated with NCBI BLAST; any contig that blasted to a mitochondrial region with score e < 0.001 was considered a likely mitochondrial region and was excluded.

We further screened the SNP library for potential outliers that exhibit high F_ST_ values among locations using the program BayesScan (Foll and Gaggiotti 2008); these outliers were included in the BeadXPress assay. To minimize potential ascertainment biases in selection of SNPs for completion of the array, SNPs with minor allele frequencies of 0.30−0.35, 0.36−0.40, 0.41−0.45, and 0.46−0.50 were equally represented. Markers with lower minor allele frequencies were eliminated to guard against sequencing artifacts as in Zakas *et al.* (2012). Further, only one SNP was selected from each identified sequence “stack” (contig) for the BeadXPress array to minimize linkage disequilibrium among markers.

### BeadXPress assay data

Genomic DNA samples at 20−50 ng/µL for individuals from three representative locations (Arica, Las Cruces, and Niebla; Figure 1) were prepared by the Georgia Genomics Facility and run on a BeadXPress platform (Illumina). Resultant data were analyzed and clustered as in Zakas and Wares (2012), with a minimum genotype call score of 0.30. More than 10% of individual DNA samples were genotyped a second time to determine genotype error rates (Pompanon *et al.* 2005) using an R script written by J.P.W. Loci with a failure rate greater than 30% were excluded from further analysis. Data were exported and basic analyses performed using GenAlEx 6.5 (Peakall and Smouse 2006).

### Population structure

Multilocus genotype data for the representative populations were evaluated for patterns of allelic diversity and heterozygosity, including standard F-statistics, to identify loci that may violate Hardy-Weinberg assumptions. Multilocus G statistics, including corrected G’st (Hedrick 2005), were calculated across sampled locations using GenAlEx 6.5 (Peakall and Smouse 2006) with significance assessed through 1000 permutations.

Data were exported for analysis using Structure 2.3.4 (Pritchard *et al.* 2000), with 10 replicate analyses of 50,000 steps with 5000 steps burn-in using sample location as prior and allowing admixture. Likelihoods were recorded and interpreted using StructureHarvester (Earl and Vonholdt 2012) to identify the most likely number of evolutionary populations using the Evanno *et al.* (2005) criterion.

### Cytonuclear disequilibrium (CND)

Data from the largest single population identified using Structure were analyzed relative to mitochondrial type (A, “northern” or B, “southern”) using CNDd (statgen.ncsu.edu/cnd), which implements the methods of Asmussen and Basten (1994) for calculating CND in diallelic loci (the statistical association between mitochondrial haplotype and the particular alleles or genotypes of an individual). Exact probabilities were calculated for all test statistics, and instances where allelic disequilibrium *D^A^_M_* exhibited *P* < 0.05 were identified.

## Results

### Specimen collection and sequencing

Libraries were demultiplexed with custom python scripts at UCD before analysis, and barcode and adapter sequence was trimmed before subsequent analysis. A total of 94,161,790 reads across all libraries were recovered for analysis, with an average number of reads per population of 3.62 ± 1.21 million in each direction. Data may be accessed from the Short Read Archive at NCBI (BioProject PRJNA227359).

### Genome-wide SNP analysis

**Figure 2:**
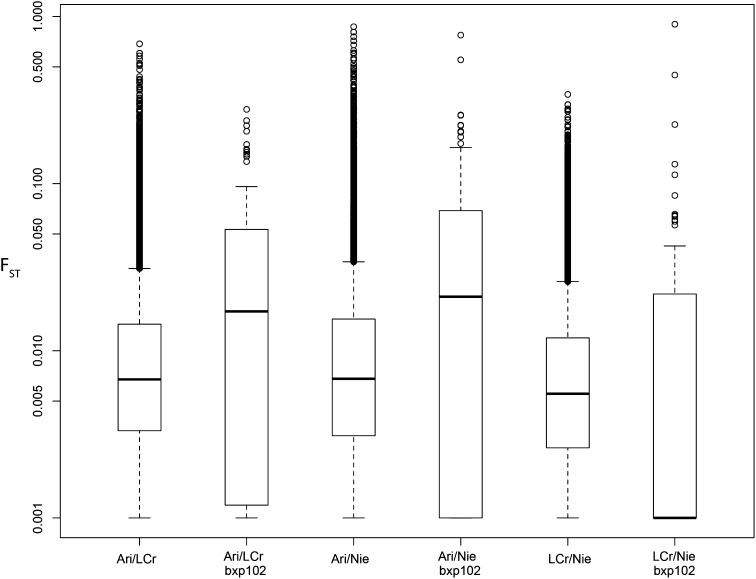
Log-distributed F_ST_ values by SNP comparing populations Arica (Ari), Las Cruces (LCr), Niebla (Nie; see Figure 1). Box plots indicate median F_ST_ for all data sets of <0.02. For each pairwise comparison, plot is shown for SNPs recovered through pooled population genomic calculation across 37,047 SNPs as well as the 102 filtered SNPs scored using BeadXPress arrays (indicated “bxp102”).

We identified informative SNP loci in two ways: first we analyzed SNPs based on groupings by mitochondrial clade (A1, A2, and B as identified in Laughlin *et al.* 2012). When these three mitochondrial populations were compared, Popoolation2 identified 58,605 biallelic SNPs that passed our threshold. Using an identical method across nine geographic populations, we identified 37,046 biallelic SNPs. A subset of SNPs was not scored for one or more locations given the aforementioned criteria. One population in particular (high tidal range sample from Guanaqueros) was excluded based on poor recovery of data in this sample. The pairwise calculations of F_ST_ between Antofagasta, Las Cruces, and Niebla are shown in Figure 2 (average F_ST_ 0.012). Results are similar for all other pairwise population comparisons, with average F_ST_ of 0.0108 ± 0.002. Results are not shown for the A1/A2/B pooled populations as our overall results indicate that grouping individuals by mitotype has no effect on evolutionary divergence that is distinguishable from spatial population structure.

### SNP selection

Of all SNPs reported, 12,339 had sufficient flanking sequence (minimum 30 nt on either side of variable site) for primer design and scoring. A total of 11,358 SNPs had optimal quality rankings using the Illumina scoring criteria. BayeScan identified 43 SNPs that had a probability 97% or greater of being a statistical outlier; in all cases these were high- F_ST_ outliers. A total of 438 SNPs had a minor allele frequency of at least 30% across the mitochondrial A and B lineage libraries. Of these, 192 were selected based on the criteria listed above for the BeadXPress array (Supporting Information, Table S1).

### BeadXPress assay data

After the removal of duplicate individual genotypes and identifying loci that met quality criteria (see *Materials and Methods*), sufficient data were obtained for 102 biallelic loci in 147 individuals (Arica, n = 37; Las Cruces, n = 95; Niebla, n = 15). Only a single individual was removed from analysis after inconsistency in genotype across two replicates; remaining data from 22 individuals with duplicate genotypes (14.9% of samples) indicated a low rate of allelic error (e_a_ = 0.007). On average, 86.67% of loci were polymorphic in these samples, with an average of 1.87 alleles per locus. Observed heterozygosity (Ho = 0.286 ± 0.009) was lower than expected heterozygosity (He = 0.328 ± 0.008), suggesting a tendency for allelic dropout, paralogy, and/or population structure. Thirty-two loci exhibited F_IS_ in excess of 0.2; average F_IS_ across all loci was 0.126. Individual heterozygosity (frequency of heterozygous loci) ranged from 30.7% (Las Cruces) to 35.1% (Niebla). Two loci (780_10879 and 63806_7593) exhibit private alleles in Arica at low frequency (<0.01 across all samples), and three loci (14707_1786, 8887_5638, and 30199_7401) exhibited private alleles in the Las Cruces sample, with frequencies between 0.015 and 0.05.

### Population structure

Global calculation of G_ST_ (0.025, *P* = 0.001) and G′_ST_ (0.046, *P* = 0.001) using the 102 loci from BeadXPress arrays indicated statistically significant population structure (see Figure 2). Eleven loci exhibited either G_ST_ or G′_ST_ greater than mean plus two standard deviations for each statistic across all comparisons. However, the greatest values of F_ST_/G_ST_ are associated with small-sample comparisons with Niebla.

Results from STRUCTURE indicated that the most likely result was for k = 2 populations, with individuals from Arica being statistically separate from individuals in central Chile. Analysis of molecular variance confirmed that approximately 3% of genetic variance partitioned between regions defined by [1. Arica] and [2. Las Cruces + Niebla], with ϕ_RT_ = 0.025 (*P* = 0.01). The latter grouping of individuals from Las Cruces and Niebla was used to test for CND, since both A and B mitochondrial lineages are found in these locations.

### Cytonuclear disequilibrium

Data from individuals in region 2 [Las Cruces and Niebla (n = 89 mitotyped with sufficient SNP data)] contributed to analysis of CND. Of 102 loci, 5 (4.9%) exhibited significant (*P* < 0.05) CND either at the allelic or genotypic level (Table 1). None of these loci contribute strongly to identified population structure, with mean G_ST_ = 0.04 and G′_ST_ = 0.083. None could be reliably annotated using BLASTn or tBLASTx algorithms (results not shown).

**Table 1:** Loci exhibiting significant cytonuclear disequilibrium between allele and mitotype (D^A^_M_ statistic and *P* value) or genotype and mitotype (statistics and *P* values shown for AA, Aa, and aa genotype arbitrarily assigned to each diallelic locus)

Locus	D^A^_M_	D^AA^_M_	D^Aa^_M_	D^aa^_M_
32209_8940	6.205 (0.015)	0.317 (0.501)	6.675 (0.016)	7.354 (0.010)
16301_4116	5.230 (0.012)	3.352 (0.105)	0.355 (0.593)	4.303 (0.062)
74853_9172	4.675 (0.038)	2.942 (0.132)	0.225 (0.792)	3.189 (0.097)
23232_3413	2.994 (0.030)	8.363 (0.012)	0.363 (0.583)	1.498 (0.271)
111068_5411	4.183 (0.039)	5.043 (0.047)	1.733 (0.289)	1.026 (0.353)

## Discussion

Exploring data that are more representative of whole genomes—and thus of the species—begins to provide more complete images of the history of populations and the evolutionary mechanisms that are associated with spatial patterns. Although the initial study of the mitochondrial cline in *N. scabrosus* suggested that it was likely to have originated via transient allopatry associated with the biogeographic boundary at 30°S (Zakas *et al.* 2009), we now see that the nuclear genome presents much less heterogeneity throughout the central and northern regions of Chile than the mitochondrial pattern would predict. Our attempt to analyze “pure” A- and B-mitotyped populations of individuals alongside geographic comparisons generated nearly identical—and quite low—patterns of population genetic variation. Interestingly, this suggests that while the mitochondrial cline centers at ~30°S, the mechanism for the strong mitochondrial divergence likely originates in regions further to the south (J. Wares, unpublished data). The mitochondrial cline documented so far (Laughlin *et al.* 2012) appears to be somewhat independent of the nuclear background found in northern and central Chile.

However, it may not be wholly independent; nearly 5% of the SNPs assayed appear to be in some form of linkage disequilibrium with the mitochondrial lineages found in *N. scabrosus*, which suggests either a complex pattern of gene flow and introgression in the past, or a more complex interaction between mitochondrial and nuclear genomes as in some other incipient marine species (Willett and Burton 2003; Burton and Barreto 2012). Interestingly, Hu (2008) showed that CND should limit the separation of neutral and selected clines, and so the extent to which these genomes are linked makes the pattern observed so far an unusual one. However, Hu (2008) also notes that selective clines often will act as asymmetric barriers to the spread of neutral alleles, a common result for introgression (Arnold 2006). Overall, this finding suggests that more information about *Notochthamalus* will be recovered from further exploration in locations to the south of Niebla; additional genotyping also will be necessary to increase the power of this test, as the five loci indicated have only marginally significant results for the CND test; correction for multiple testing would, in fact, require a *P*-value of <0.003, which none of these loci obtain.

Nevertheless, these results confirm the initial conclusions of Zakas *et al.* (2009), which showed population genetic homogeneity in the nEF1 gene region. Here we find that although there is statistical separation of genetic data from a site in northern Chile (Arica) to one that is over 2,000 km south (Las Cruces), the actual level of nuclear differentiation (G_ST_ of 0.025) is small relative to the ~2% sequence divergence and reciprocal monophyly between the two mitochondrial lineages. The thousands of SNPs from our RADseq data support this inference of nuclear homogeneity in central and northern Chile populations of *N. scabrosus*. Practically, these results also suggest that—for experimental and community ecology in the intertidal—there is little reason to treat the two mitotypes as distinct species potentially in competition (Shinen and Navarrete 2010; Laughlin *et al.* 2012) across the ~3000 km of coastline considered here.

Our results from analysis of SNPs from RADseq data and direct genotyping via GoldenGate (BeadXPress) technology generated comparable patterns of allelic and genotypic diversity. These results compare favorably to those of Gautier *et al.* (2013b), which note that pooled next-generation sequencing libraries generate comparable results as individual-based analyses, with lower cost and complexity. Still, given concerns about the potential bias in estimates of genetic variation from RADseq data (Arnold *et al.* 2013; Gautier *et al.* 2013a), it is important to weigh the potential for bias in these data prior to use in larger-scale analysis.

The enigmatic discordance between the mitochondrial cline in *N. scabrosus*, in which a highly divergent lineage appears at about 30°S and increases in frequency dramatically in populations to the south, and the nuclear genome—which exhibits relative homogeneity across nearly 3000 km of coastline, is not a typical case of divergence in the nuclear genome lagging behind that of the mitochondrial genome because of larger nuclear effective population size (Edwards and Bensch 2009). For a similar mitochondrial divergence pattern in the confamilial barnacle *Balanus glandula*, spatially concordant nuclear divergence is exhibited in multiple loci (Sotka *et al.* 2004; S. R. Palumbi, personal communication). In *B. glandula*, both mitochondrial COI and nEF1 loci exhibit a tight similarity in latitudinal transition from northern to southern lineages. With similar data from a confamilial species along a large portion of the Chilean coastline, Zakas *et al.* (2009) could not reconcile the lack of phylogeographic structure in nEF1 relative to the strong transition in mitochondrial COI. With the data here representing a much larger number of markers from the nuclear genome, it is clear that either the mitochondrial cline represents selection on divergent mitotypes, which has been observed in other marine systems (Schizas *et al.* 2001, 2002), or the phylogeographic diversity of the nuclear genome will be clarified with sampling of individuals further to the south than in this study, or both.

We have recently discovered that the southern mitochondrial lineage is fixed for the B mitotype in populations at the southern end of Argentina (Wares 2013; J. P. Wares, unpublished data). It is likely that the region between the southernmost samples represented in the current data set and our collection from southern Argentina will identify further diversity, and perhaps divergence, in the nuclear genome. Determining whether the spatial discordance of mitochondrial and nuclear diversity is a stochastic result of independent loci responding to neutral mechanisms, such as migration and drift, or whether there are fitness consequences for the apparent linkage, requires further exploration of genomic diversity across the full distribution of *N. scabrosus*. The current geographic sampling centered around the 30°S mitochondrial cline limits our ability to interpret historical and ecological effects, considering the full species’ range is distributed across >5000 km of coastline. The apparent mitonuclear decoupling in these coastal populations presents an intriguing pattern that may involve distinct oceanographic and environmental mechanisms influencing different genomic partitions in *N. scabrosus*.

## Supplementary Material

Supporting Information
