# Supplementary material for: Homogeneous Nuclear Background for Mitochondrial Cline in Northern Range of Notochthamalus scabrosus
Source: G3 (Bethesda). 2013 Dec 17;4(2):225–30. doi: 10.1534/g3.113.008383 (PMC3931557; doi:10.1534/g3.113.008383)
Supplement: Supporting Information [file supp_4_2_225__index.html]

Homogeneous Nuclear Background for Mitochondrial Cline in Northern Range of Notochthamalus scabrosus — Supporting Information 

# Homogeneous Nuclear Background for Mitochondrial Cline in Northern Range of *Notochthamalus scabrosus*

## Supporting Information for Zakas, Jones, and Wares, 2014

**Files in this Data Supplement:**

- Supporting Information - Table S1 and Files S1-S2 (PDF, 675 KB)
- Table S1 - Oligos selected for Illumina BeadXPress array development. (PDF, 87 KB)
- File S1 - Full genotype data from 102 SNP loci in the barnacle *Notochthamalus scabrosus* in GenAlEx format. Nucleotides are encoded as follows: A - 1, C - 2, G - 3, T - 4. (.xlsx, 147 KB)
- File S2 - Major allele frequencies at 37,046 single nucleotide polymorphisms identified through RAD-seq methods for 10 geographic locations of the barnacle *Notochthamalus scabrosus*. (.xlsx, 3 MB)
