# Supplementary material for: Homogeneous Nuclear Background for Mitochondrial Cline in Northern Range of Notochthamalus scabrosus
Source: G3 (Bethesda). 2013 Dec 17;4(2):225–30. doi: 10.1534/g3.113.008383 (PMC3931557; doi:10.1534/g3.113.008383)
Supplement: Supporting Information [file supp_g3.113.008383_TableS1.pdf]

Table S1. Oligos selected for Illumina BeadXPress array development.

Locus Name	Alleles	Sequence
73099_2932	C/T	AAACGGAACTTGTCTACCAAGGAACACACAGG[C/T]TCAATGTTTCGGATATTCGGCATCGATTTCAGAGCGAGGAAA
23164_2652	C/T	AAATTTAACATTAACCCTAAGGCGTTGAATGG[C/T]AAGAAACGTGTCGAAAATCACTCGAAAACCACTTTCGCCCCC
9187_9477	C/T	AAATTTTGTGTTTATATCTTTCGTATTTATGAAGATATACG[C/T]CAACAAGATTTCAATCCTGGCTGGGTTGTGTCG
85340_9202	T/G	AACAAAATGTGCTGCAGATAAAAAGGCAATAGCCTGATTT[T/G]CCATCGCACGTGACATCAGGAGGCAAATTGTAAC
32209_8940	C/T	AACGTGCCCGAGGCTGACCAGATTGCAATTATCGTGAGTT[C/T]CCTCGTCAAGTGGTCACCTGTGTCGTCAGCTGTT
56086_538	C/A	AACTTACCAGCGTGCTCAAATGGCCCCCA[C/A]TTTCCCCTCACCCATGTCCGACCGAAATCCAAATCGAATTTTGCA
45100_11302	T/C	ACAAATTTGACCAAAATTTTACCAGCAGCGGCATCACGACGTG[T/C]AGGTCATCCCAGCTAAAGCCGGACGACATTC
20748_8065	G/C	ACACCTCCGCTTTGCAGAGCTGCCTGACAGACTATGTCG[G/C]CAAAACGCGAATAGGAAAAATACCCATGCTTATAT
3858_3953	T/C	ACCATTTTGGTCAAGTCCGTTCTAATATTCACCT[T/C]GTTCACGCTGCTCAGGATAACTTTTTACAACACGTATCGG
56743_5204	C/T	AGAAACTCATCCAATGGTCTCAAAGAGAGGCACAG[C/T]ATTGATAAAATCCGCGATTTACTGCTGAAAAATGGTTAC
29174_1159	C/T	AGAAGGGGGGTCAGAGTCCGTGTCATCTTT[C/T]TTACGTTTAGGGGGCTTCCCCATAGCGGGGAGCCTTAAACGGAA
35574_5065	C/A	AGATCGGAAGAGCACACGTCTGAACTCCAGTCACT[C/A]GCGTATCTCGTATGCCGTCTTCTGCTTGAAAAAAATTTT
70470_5281	G/T	AGCGTCGACATGTTCCAGAGATTCTTCTGGGGCAT[G/T]GGACACGTGCTCCTCGACATGGTCAACACCAGCTTGACA
24338_6587	C/G	AGCTTATCCGTGATAATCCCCAAACATCATATTTCGT[C/G]AACATTCCATTCGTTTTGCGACCGCCTCGGGCAAATG
780_10879	G/A	AGTGAAACTAATTCCACTCTTGAAGGCTTCATAAGTTTACATT[G/A]GCGTAATACTGAGACATTGCCGACAGGTGAA
78971_9936	G/A	ATAGTGACGTTTACTCTCATGAAAACAATAATCATCGATTT[G/A]AAAGACATTCAGAAAGTTAGGACTAACTATGTG
98697_3006	C/T	ATATGATACGCAGTACGCACCCCCTGAATTCG[C/T]TGGTAGAGTATTTTGCCTTCGTACACCGTAGGTATTTTTAAG
63483_5247	T/C	ATTAGTTTCGGAGATAATCTGGGGGGGGCACTTTT[T/C]GCCCCCCCTCAGTCAATGGTGTTCTGCTGAGACACCAGT
42103_2016	A/G	ATTAGTTTCGGAGATAATCTGGGGGGGGGGC[A/G]CTTTTTGCCCCCCCTCAGTCAATGGTGTTCTGCTGAGACACCA
54648_2087	T/C	ATTTCGTACCCTTCAATCGCATATCCACTGA[T/C]AAGTAAGGGGTCCAGCCACGTCTCCGATATTAAGATAAAGTCT
17922_11098	A/G	ATTTTGCAACATATGTTGATATTGCAAAAAATGGCCAATCCTG[A/G]CCAAATTTGGTCACCAGTTTGGCCATGAAAT
17477_1045	G/A	CAAGGTCATCCTAGGTCAAGGTGACATGTG[G/A]CCTTGACCTAGGTCAGATGAGGTCACCAGAGCCATCTGGTCATT
92849_676	A/T	CAGAAGCACAAGTGATGTGACCTAAAACG[A/T]GGTCGTTATCACTGTGACAGCTAATTGGAAATAATCGTCTTTCAC
7306_10222	T/G	CAGTCTTAAACGACTCTCGCACTATTGTAAAACCCAAAACTG[T/G]CGTAACCACAGGCCAGGTGAGCTCCCGATATG
69655_10675	T/G	CCATGGGCTTATTATCAAACCGATTTGCACCGGACAGACACA[T/G]AATTGATTGTAGTTTATGGTTACTGAAACACA
29868_5022	G/A	CCCACATCGAACAGTTGGAAAATGCACGAATTAGC[G/A]GTCTTTAGTGCAAAGGTACGCCAACTGTCAACGGCCTTG
3236_3937	A/G	CCCTGGCGTTCCAAGGACTCGGCGGTCTACGGAC[A/G]TTGTAAGTGTCATCATCAATACAGTTTCCTCAGCACTTTT
42122_2784	T/C	CCGCGACGCCTGAAGGCCGGAATTAAAGGCAC[T/C]CCACAACAAGCTCAGGTCGCCAAAAGCGTCCATCATTAACTC
62442_7584	G/T	CCGGTACATAACTTAATCGTAAGCCTTACCATTTGAAA[G/T]TAAAATTTATATTTTTGCGCGCTGAAGGCGCAATAA
28801_360	C/A	CCTTAAATGTCGGAATGGGTCCTCTGCAA[C/A]TCGAAGTGAGGCCCTGGATGTCACAATGGGACCACTACAGCTTTA
25964_4994	T/A	CGAAAATGTCAAAATATCAGGATGTCCAATTTTTT[T/A]AAATTCCTAACTAAAAATAGCAAAATGCCATCATCACCA
62506_4434	T/C	CGCCAAAGCAGTCTGACCAGTTGTATTGTAACGC[T/C]GCCAGGTGTAAAAATCAGTCGAGGCACAGCCCGGTGACGT
116107_10040	A/G	CGCGGCAGACCATGCAATGATGTGGGAGGCATTTATAAAAT[A/G]GGCTAATGATTGCATTATGAAACATGCATTTTC
10150_4848	T/G	CGTCGGAGAAAGAGTATGGCCAGTTTCATATGTTT[T/G]CGCGACATTTCCAGAACGTGACACATCTACTGGAAACTG
22375_8872	G/A	CTCTGAAATGCATTGCTATGTAGATGTATCATAACAATAG[G/A]TTGGTGTGTGTCTTCAAGGTCATGTGAGGTCAAA
48724_4366	A/G	CTCTTTGCATGTTTCTTTGCGAACAGGTAATAGA[A/G]AGTGTGCAAATAAACTCTTTCATGTAAATAGATTGACGCA
74853_9172	T/A	CTGCTACCCACTCAGCACACCCATGGTCAACATAGGAAGT[T/A]AGCAAACAAATAAAATGAAACACATAAATATAAT
5269_6372	T/C	CTGGAAGCCTTTCTTCCGGTTTATCAGGTAAGTGGAC[T/C]AGCTGAGCAAGGTTCCAAAGCATTCAAGCCATCATAG
12787_9519	C/G	CTGGTCGTGCCGGTCGGCTGCTTCTTCGCCTCCAGGAGCCT[C/G]CTGTTTGAGCTGGTGCTGGGCCTGGACAGCAGC
19979_8853	C/T	CTTCGGCCAAGTTCCCTCATGAAGGGAATTGTTAGTTGGC[C/T]AATTACACCGGACGTTTCAACGGCGACAGGTTGG
14448_11062	T/G	CTTGCCATGTTGATTAAAGTCGTCAGATAAACACCTGAACTGT[T/G]CCTACCCAATCCAAAATGTCAACCATTTGCC

12131_2534	C/T	GAAACATTGTTTCAATTGGAATAACATTTTCC[C/T]ATTCTTCGCTATGTTTATCCTTTTTGACAGCTTAAAACCGTC
112279_4586	A/G	GACTGAAAAGCTGCACGGCTGTAACCGTGGAAAC[A/G]ATTATGTTTGGCGCCGGGACGGCTGGACCAGTCACGGTTG
94670_5362	T/C	GAGACAGCATACCATTACAGTTATTTGTAGTCGCA[T/C]GTCGGCGTTCGTTTGTTCGTTTATAGGGGTTTCGGGGCG
27196_8125	C/T	GCACCCATATTCTAATACACTCTCTTGGCGGAGCAGTTC[C/T]AGAGGGTGTCAAAATCTCATTGTCGTGGCCCACGG
16967_4125	T/C	GCAGTCCCACCTCGACCCACAATATTAACCGTGC[T/C]TGTGAAGACTGACGGAGAGAGAGGGGAGCAACATTGGCTC
14348_2559	C/T	GCCATAAAACCTACGGATGTTTGAGTGCGGCG[C/T]TTACGAAAAAAGCCGCGCGCAGCGGGAATAGAACCCGCAAAC
15866_3332	C/A	GCTGAGTGAGTCACAGACGGAGTTGAGTCGACT[C/A]ATGTCGAAAAATGTCAAAATGAAGTAAGTTAATTGTTTACT
141858_10826	A/T	GCTTTTATGTAAAGCGCCAGTTTTCTTAGAGGTGTTGTTTTT[A/T]AGACTCGAAAAAAATAATAAATAGTATTTGCC
31608_11985	C/T	GGCATATTTCAACATTATCTCATACCGTGGCGAATTATCTCATC[C/T]CGTGGCGATATTGAAATACAACCACAGGCA
25108_4214	C/A	GGCGTGCAACGTTCCGAACGAATTTATTTCATGC[C/A]TCCAAGGCATGACAAACGTGGAAGCGGAGGAGCTAGAAGG
79212_12235	G/A	GGCTGAACCGATTGCGTTCAAATTTTGTGTGTGGTTAGGAAGTC[G/A]GGGACGGACCACTCAGCCGCCAAAGCGTGC
36824_8982	A/G	GGGATGATTTGCGTGTGTTTGCAAACGAAATGAACTGAAC[A/G]GTCAAATTCATGCTCACCACCATAGGCCCATATT
48018_2052	C/T	GTTTTCTAATGTCCGGGTACTTTTTGCGGTG[C/T]TTCTCCACCGCCTGGAGCAGATGTTGCCTTTCTGTGTCGTCTT
10217_6433	G/T	TAAATAACCGCGATATCATCAACCATTGTGTCAATTC[G/T]TTCTGTGACTTTAGCCCGCATCATTTTATCCGCGTTT
10579_2515	C/T	TAACACCAATATCCTGTGTCCTTCAAATCATA[C/T]TTTGTTTTCCATTGGTGTCACTTTTAGACAGATTACGTTTCT
6440_10204	G/C	TAACAGCCACGGTAAATTACAACACGTAACAACACGGAGGTG[G/C]TCAAAACAGCGATAATTTTCCGCAGGTGAAAA
19653_8054	G/T	TAACTACTACCGAACTACCAACTGCTCCCGAACTACCGA[G/T]GCTGTTACCCGAGTATCCGAGCAGGAGTCCCATGT
41462_9749	G/A	TAAGGTGCCGTGTTCGGCGATGAACGAGCGAGCGCGAAGGT[G/A]CGTGGACTGGCGTCTGAGTGATCTGTCCCTAAC
85901_8414	A/G	TAATTAGGCCAAACAACCGAAGAGCGCAGTTCAGACTTA[A/G]CTTGGTCCTTTATTTGTCTTGCGCTTAATAACTTG
23254_307	A/T	TACACCAGACAAAAGTGACCGGCCGGTTC[A/T]TGGGAAATCTGATACATTTCTGGAGCAACTTGACGTGCTCTGTGC
17469_1817	G/A	TACCCCGTTTGTATGAGACAAAAGTGTTATT[G/A]CTAAATACCTGCCTAAGTTTTTCTGAACGCTCTAAAATATATT
8887_5638	G/A	TACGACGTTTTAACAGTCCATCCTCCATGAACAAGA[G/A]CGGTAACTGAGCTGAACTCAGGTAGAACCTCAGCTCGA
21120_11884	A/C	TACGGTGTCCGTCAAGGACTCAATGTTGAGGGGCGGCCAAAGAC[A/C]TTCAGAGGATGATAAAGATGTTCCTTGATT
74262_5296	C/A	TACGTGCAACTTTGGCGCGCACACACTCGTACTTG[C/A]TGTGATGTTTTGGCACAATCCATACTCAACCAGTGCAGT
18148_1823	A/C	TAGCCTCGGCTGCCAGTCATTCTCTACACGA[A/C]AATTCGTTTATCCTGAAGTCTGCTCATTCAAATGTAAACTCGC
45792_7502	C/T	TAGGCAGCTTATTCCAACGACGTGGACTCCGTACGAAC[C/T]CGGAATATGGACGATTCCCTTTGACAAAAGCACTGA
16301_4116	T/C	TAGTACAAGTCACGGTAATGGAGCTGAGGTGCAG[T/C]ACAATGTAACCCATTAAACACTTTCGCACTGCGAAAACTT
99732_9250	G/C	TAGTTTAGTACTAGAAAATATTAAAAATTCTGCTATGTTT[G/C]ACTACCTCGTAGTAAAGCGTGTAAAAATCGGAAA
83445_6898	C/A	TATACAATCGGTTATATATGTTGACATGAAAACAGTC[C/A]TTACCATCGACGCACTCACGGCATTCCGAGAGATCTG
16596_5733	G/A	TATCTTTATTAGTCCTGCCTCATGACCCATATTCGG[G/A]AACATATAATGTCGAAGAATGTCTAGATACCAATGTCT
48348_6758	G/A	TATGTAAAACTGCAATGGGCGGCAAGGTCATGCCAGT[G/A]ACGCCATTTGTAATCGAATAGCGAATACTATTCAATA
48348_11327	A/G	TATGTAAAACTGCAATGGGCGGCAAGGTCATGCC[A/G]GTGACGCCATTTGTAATCGAATAGCGAATACTATTCAATA
34436_3503	A/G	TCAACTTAATACTGTACCGAAATAATTGATGAA[A/G]CGACCTAGGTTCTACAATGTACTTAACTACTACATTTTTTT
17471_1818	T/A	TCAATAGCCAGAATTCCTACATCAAATGGAG[T/A]GCAATGATGCAAATATGAACAAATTGCAACCAAATTATGCATG
76636_4487	G/A	TCACAGACCTCCCAGGGGGTGGTGCGCCACAGCA[G/A]GTTACGGGGCTGTGGAAGTCACTGGTCGACATAGTGCAGA
42061_9753	C/T	TCACGTGACATTGCCTTGGGTGGCAAGCATGTCAATTTCTA[C/T]CCTAATGTCCATAAGCTGGAAGGTTCCAGCTAT
10768_8742	A/G	TCACTGTCATAAACCAGCTACAAAAACAATGATATGACCT[A/G]ACTGTCCTTGCAGTTTCTCAGTAACACAAAACAC
77381_2945	A/G	TCACTTGCACAAACAATAAACTTCAGCCAACA[A/G]CGATATCTACGCGGCTGACATGGCAATAAATGAAGCCAGTAA
36666_10486	T/C	TCAGAATTGCAATTATACAATGCTGAATTGCATTCGATGCCT[T/C]CACTCGAAACTCGAAACACGTGCTGTTGCCGT
81022_2957	C/A	TCAGACATGTCGCTACTCTACTCTACTCTACT[C/A]TGTGGCTACTTTGGAACTGTTTCAGAAATCAGAACTAACAGG
20805_4958	T/G	TCAGGAATGTGGCTTGGTATTCATCCTGCCTCACG[T/G]GATTTCCGTTTAGTTTCTTTTAGCTTTTACTTGTTCTTC
90914_1449	T/A	TCATAGATGGCGATGTTGCAACCGATTATA[T/A]AATGAGGGTCTTTTCTGGACATATTGCTTTTTTGTGAAATAAAC
63710_9871	A/T	TCATAGGCAACACGCTGGTTTTTGTTTCGAGGCTCTAGCTC[A/T]ATTACAGGCCGAGTAACAGAAGAAGAGGTCGGG
26036_338	T/C	TCATAGGTCGTCCATAAACTTACGTTACA[T/C]TCCTCCACCTTTTGCTAGATTTTCCACTGTTTCCCTGCGTGTGAC
91805_9973	T/C	TCATCAGAGCAGTGCTGAGCGGCCGTACCAAGTCACCAGAG[T/C]AGTGCTGAGCGGCCGTACCAAGTCATCAGAGCA
42084_9754	T/C	TCCAATGGATTATCTCCGTGAATCAACTTATCCTAGCTTTA[T/C]ATCTCCCCTTACTACCAATCCAGGACAAATAGA

37230_1983	G/T	TCCCGACTTACGTGGGTGGCTATCACTGCGC[G/T]TGGCACGTGCCGCGTGAGGGGTGTTGAAATACTTTTTCTCGCA
63806_7593	C/T	TCCGACATGTTAAGCCGCTTCACCTGTATGCTCTCGTA[C/T]AACAAGTTATGCATCACGAACGAGATATTCTTCTGC
36553_11250	C/G	TCCGTTCTACCGATAAATGAAAAGCCCTATTTCATTTTGGACA[C/G]ACCTTGTATGCAACTTATATAATTTGGTGAG
24435_7353	T/C	TCCTTACTCTGCTTGTCTTGCTTTTTTATTTGTTTTGT[T/C]TCAACTTCTTAGCAGGGAGTGACGCGACTTGCTTTT
34954_1207	A/G	TCGAAAAATTGGTTAGTGCGCAGCTGATGT[A/G]TTACCTCACCAAAACTCACGTCATGACCCCGACGCAGTACGCGT
88409_12268	C/T	TCGCAGAGCTCGGTAGTAACAAGCAACCACGAGAAGTGTCCTGG[C/T]TCGTTGAACACGGTCTGTCCCCGTAGAACG
78133_12229	G/T	TCGGAGCATTTCGGCGTCACATACGTAGTATGTAGGATGTAGGA[G/T]GTATGCATGTATGCATGGATGTAGTATGTA
115103_2274	G/A	TCGTAGACAAGACGCATGTGAGATTTTGCTC[G/A]TAACTGTTTTTGCGCAACGCCATTTGCCGCACTTTCACAAGAA
45017_4347	C/A	TCGTCAGGCTTGCGCAATGCGCAACAACATTTTC[C/A]CTCCACCAATGTATCCATATCACACCGCGTCCACCGTAGC
43731_1265	G/A	TCGTGCCACCTTGCACGCTAGCCTAAGCTC[G/A]GACAGAACGGACTGAACACACAGCATAGACAGAAAGAACAGAAG
22514_3409	T/C	TCGTTTGAAGTGTTTTTACACGCTCAGTCGTAG[T/C]GAACTTGAATCATTTTCTTTGACTAAAAACATGTTTGTCCA
84850_3741	A/G	TCTATCGTCTTTAACTCAGACGTACTGGGGGTA[A/G]ATTTTCCATTCAGCTAACTTAGCCTTTATTTCTAGGTTGTG
44122_12075	T/C	TCTGAGACAGATACCACCTCAGGAGTGTGTAAGGGTATGATACA[T/C]AGTATCACAGGATATTACAGGATATACATC
87102_6157	G/A	TCTGGCGAAAAGGGTCATTGACACCAGCCAAGGAAA[G/A]TATCAGATACAATGGCGCGCCGCATGTATGGCATTGCA
5401_3972	T/C	TCTGTTCGAAGGGGCATATTTGTCTACGGGTGGC[T/C]TATAAATCCTTAGAAACCTCATGATGCATCACGAGAAGCA
16666_3340	A/G	TCTTTCTAGTACTTTAGATAGAGTTGGGAGCAA[A/G]CTTATTGGTCTGTAATTTTGAGGGTTAGAAGCGGAACCTTT
62727_7587	T/C	TGAAGCGCCGGAGTAGCTACGTGGTCACTGAGCTCAGT[T/C]GCGGGGCAGTAGCATGGTCTTTGGCCACTGTTTAGT
28715_5849	G/A	TGAAGCTCTAGATAACTGAGATTTGTTTTAAACGAA[G/A]TGATACATTTTTAACTCTCAACATGAGCACACTCATCA
1066_3902	A/G	TGACAAGTTTTTCAGAAAAATGACGCCATTTTGC[A/G]GTATGCGGCATGGTGTGGTGCGCCATGAGCGTCACGAGTC
71757_1380	T/C	TGACACAGCCTACTACACATGGATACAAAC[T/C]ACATTCCCACATTCAGTCATAATTTGTTTGCGCCCCTTCGGTGT
9180_4834	G/A	TGACATGCTCTCACATAGTAGGACGGCGCTCGGGC[G/A]GGCTTGTCCTCAATTAAATATGCGGTCTATTCGTGGAGC
37735_10495	G/T	TGACTAGGAGATATCCAAACTGGTCTGCCATGGTGTGGAGTC[G/T]GGGGTCCTTTCTGGCGTGGCTGAGACGGTAGG
10278_4039	T/A	TGAGATACAAGTGGGTGCAAATGTAGTGAGATGG[T/A]CTGCAGAGGTTCTCTTTTGGTGTAAAACATCATTGTAATA
88585_1440	T/C	TGAGGGTTTTCAGGACGCATATAATTCCGA[T/C]ACGAAAGAGCGGAGCGTCCCTTGCTACGCGAATTGCCTAGAACT
61336_4429	G/C	TGCACAAGCACCCTTTTGAACATGCTGTGAATCA[G/C]CAGAACGGAAATCAGGGCGAAACTGCGTGCCGCCTCGGCG
20406_3390	C/A	TGCACATACATATACCTAAACGCATTTTGCCAG[C/A]AGTTTTACGGGTAAGCATTGACAGGGTCGCAAGTATGTATG
18173_1057	C/T	TGCACCCAGCGCGGACGCGGACGCCATGAA[C/T]AGATACTTTGTGAGCATAGGTACCGCGACCGCCCGCCAGGTGGA
19597_3385	A/G	TGCAGGCACGTGCCGTTATGTTTGAGGAATCTA[A/G]TGAGCTCATACAAAACCATTTTGCACGCATGCATTACCTAC
42277_5951	T/C	TGCAGTAAACTATGCAGATACGCAAATCAAGCTGTA[T/C]CCATAGCCACTAGCAAGGCCGAGGGCCAAGTTTGCAGT
23232_3413	C/T	TGCAGTGAAGTGGTGGGTGCAGGGTTGTACACG[C/T]CAATCTTTCAAAATAGGCAATGCTACCAAATGGAACAATTT
57816_2873	A/G	TGCATAGTTTACATACAAATCTACATAGCCCG[A/G]TGACAACTTACACGTGCGTTTTGACCCTGTTGGCATCAAAGC
72541_10683	G/A	TGCATATGCCAAGCTATTTTGTGATAAAGTTTTATGTACATG[G/A]GCCCATTTCATAATTCAACTGCATGTAACTTC
29591_1161	G/C	TGCCTCCCAGTGCTGCCGTCACAGGAATGG[G/C]ACCTGGCCGACTGAATAACACAGGCGCAGTCGATTAATTCGTAA
6584_7166	G/C	TGCGCTAAGTAGTAATGGACAGATAGAGGCACAAATGG[G/C]CCCTAGGCAGCAATTGTTAAGAATTTCAAACTTTGT
6584_10207	A/T	TGCGCTAAGTAGTAATGGACAGATAGAGGCACAAATGGGCCC[A/T]AGGCAGCAATTGTTAAGAATTTCAAACTTTGT
30294_1168	C/T	TGCGGAGTGGCAAGCTCGGGCTCCTCAAGA[C/T]GCTACGTCAGCGTGAGACCTTGGTAGGTCGCTGAGCTGTGCTGC
3199_9399	C/G	TGCTAGTTTTATTCTAATTAATACGAGCATGCAACTAGGAA[C/G]TTCCAATGTTTATGAACACCCCCAGTGTATGTG
109776_12318	T/C	CATGTAGTGCGAGAACAGTCCCAGAGTCCCTTTCAAGTACGGTC[T/C]CGGACCCTCCCACTCCCACCGATGACCCCC
111068_5411	A/C	TGCTGCTGCTACACAGCGCTGGCCACATGAAAGGC[A/C]ACCCTTTGTCTTGACGAATTTACAACATGGCTCACCTAC
73865_12216	G/C	TGGAGGTGACTTCTCCTGTGTGCTCCATTTGTAGCAGCAAGCAG[G/C]CACAGTACAAATGCCCAATTTGCAGGCTCC
26612_3435	C/T	TGGCATTATTGGTTGGCAGGTTGGATGAATTAT[C/T]ATCCCGGATTATCAAGGTGGCTTTCCTTCCTGTCCCTATAT
53469_9078	T/G	TGGCGACTGCCCATGTTAACATAATTGGGAAAAACATCGT[T/G]AATTTTGTTATGGCAGTGAATGATTTCAATACAT
9076_4027	T/C	TGGCGCTAACCCCGCTGGTTCCGCTCTGTTTCAG[T/C]CCCTGTAACAGCGTCTCGGACAAAGAGGAGAGCTTCGAGT
33831_3499	G/T	TGGCTTCCAGTCTAAACATCGAGGGAGCCTTCC[G/T]TGCTGATGGGTTAGGTCGCCATAACCCCGCGAATTCGTCTG
30199_7401	T/C	TGGGAATTATGGAAAATAAGTTTCAGATACCTACGTTT[T/C]CAGGGTCTCTCAATCTATTAGCTGAGCATGTCAGCC
121397_2284	T/C	TGGGCATGTTCAGCATCTGGAACAGAAAACA[T/C]CGGGCGTTAGTAGTGCGGTGGATTATATGAACCCGACGTTGTT

10472_2512	C/T	TGGTGCATCGTGCGCTGACGACCGAAATGCGC[C/T]GACATCGGAACATCGTGCGCTAACTGAATAATGCGCTGCTGC
5820_2438	C/T	TGGTTCCGACTTGACACGCAACGTTTTATGCA[C/T]GTGGATAGACGTGACACCGCCCACTTTCCAACGTGGGCTCGT
46614_11314	G/A	TGTAAGTAATTGCTCATAGGTATTTACACGAATGGTTTAAAAG[G/A]ACCATCCAATCCAAGATTTTACTGGGTGGAA
17099_8025	C/T	TGTACAGTAGAGGCCCAGATTAAAACAGATGCATTGTTT[C/T]GGAACATAACGTCAATGTCGTCAAAAACGTCGCCT
40382_5935	A/G	TGTATGCCTGCGCTTCGGTAAGCGAATGTCCACTTG[A/G]GAGTTGACGTTTTTTGCTACCCGGACATATCTCACTTC
24547_325	C/G	TGTATTTTATGAGCATCGGAAACTCACTC[C/G]GAAAAAAATTATAGGGCATTTTTATTTTATGAGATATTGTCACCA
132654_3836	C/T	TGTCGCAGAACACCATTTGACCTGTGCCTGTAA[C/T]CCATGTGGGTTAAACGGGGTCAAAATGGTTAAAAACGTCTC
77736_11447	C/T	TGTCTGAACCAGTTTTGTGTACGTGCAAAGACCTGACCAATAC[C/T]CGATGGTGTGAAGATGTGATGATTGTGATGC
17849_3356	C/T	TGTTCATCGCGGCATCTACGAATAACTTGAGTG[C/T]GGATAAGGAAGATTGATGAAAAATCAGATAAAATCAAGAGC
74571_2936	T/A	TGTTTTTCAACCATCCTTTGGACGCATAGTGT[T/A]AATAATCGCATTTTTGCGCTTTCAATGGGCGAGGAAAAAGGA
54583_5195	G/A	TGTTTTTGAAGTGGAATTGTCATGTAATTGATACA[G/A]TTAAGTGATGTGTGCAATTTCTGAGCTAGTGGGTCGGCT
55334_3636	A/T	TTACACGACGCAGGCAAGACAATGATTGAATAC[A/T]TGTGTGCAATGACACTTCAAATAGTCGCTTTTAACACGGGA
31507_8154	G/A	TTAGATGTATGTTAATCGACACCAGCTTGAATAGCCAGA[G/A]TCATCTGGCTGAAGGTATAGCAGCTTGACGACATC
89626_7674	A/G	TTAGTAGGTACGCACTATGCACTATGCAGGTATCTATC[A/G]AGGCAAACTGGCAACGAAATTGCACGTGAAGAGGAG
101689_2253	G/A	TTAGTCAATTGCACATGCCAATAATGATAAA[G/A]CATCACATTAACTTTAAGTTCTAGGCAATTCGCGTAGCAAGGG
20383_7309	G/T	TTAGTTCTTTCCTGAAACTTAACTAAAGGACTAAATTA[G/T]ATTAGAAGATTGAAATGATTTGACACTATCTGTTAG
171191_3087	C/T	TTATACGGTTATCACTAATACTTCAACGGGAA[C/T]AGCAAACACGAGCTGAATCGAATGCTATCACCGGCCAATCCA
48036_9042	G/C	TTATATCTGCAAGTTTTCTGGGGTTAGCCAAGCACAGCCG[G/C]CATTTAGTATAGGACTCGCACCAAAAGCTTTGCT
14707_1786	G/A	TTATATCTGCTCTGAATTCTGCTATCCGGCC[G/A]GGTATCGGATAGGCCGGATAGTACCAAAAAAGCTAATTGTCTG
103030_9256	A/T	TTATCAGTTAAATTTGCACCATATGAAACTTAAGGATGAG[A/T]AGTTTCATCAAGGTTGCGGGAGCTGGGGGGCAAC
45415_7499	A/T	TTATCTCTAGTGACTGCTGTAGTTAGCAACCAGCAAAA[A/T]ACAAGTGGTGACTGCATCGATGAACAGCACACTATC
700_811	T/C	TTATGATGAATTGAGCGCAACGAATTCGAG[T/C]TCTTTATTTCTTCTTCCCCTTCTTCTTTCCAGCTCCTTGCCATT
27789_353	A/G	TTATGCGGTTTTATATCTACTTTAGATAG[A/G]TGTAAAAAAGTTTTCAGTGTATGCTAAGAACCCGATTCTCCGATA
15134_225	T/G	TTATTGCCCCGGAACATGCTTCCGTATCG[T/G]ATGATGACCAGTCGGTCTTTGACGGAGATTCCGAGCTGTTGCAGC
22164_9613	G/T	TTATTTGGGTGAAACCACTCCTCTAACTGAGAGAGAATCAA[G/T]CATAACTTCCCAAAATTAAACCTTTCGTTTAGC
81903_6892	A/G	TTCAACAACCAGTGCATACACAAGTTCGTTCACTAGC[A/G]CTGCCACTAGCTGTAGGTACACTGTTTCGCTAGTTTA
2710_10151	A/T	TTCCCCCCTTAAGCGCGGAGCGGCCAGGTACGAACACATCAA[A/T]AATAAACGAAGTCCGGCTGTGGCCGGAAGCGC
19656_1846	A/G	TTCCGACGGCATAAGCTTACTGTCGGTTACC[A/G]CTGACTGCAGCGTGCTGCTCTATACTGGCCGACAGCACACCGC
1672_7091	A/T	TTCCGTCAGTAAAATCTCTGGAGGCTGGAGTGTATGAA[A/T]GCGAATGTCACACTTGAGTAAGCAGTAAGCAGAATA
112208_6221	T/C	TTCGACCATGAAACACGCGTGAACACGCCAGACGCA[T/C]AATTGCGTATTATTTATGACATTTCCTGCGCCATACCT
27766_9652	T/C	TTCTAAACCAACAATTTTCAAAAAAATAATTTTGACTAAAA[T/C]AATGCATTTGGCAAAATTTCAAATCACTTTAGC
54109_2084	A/G	TTCTGAATGTCGTTGAACTGTTTTGAGAAAT[A/G]CTGAAGTATCTTTACACAGACAGTTGTTGGGAAGTTTCAATAT
20401_6550	A/G	TTCTGCTCGTCGGTCAGCAAGTGAAGTAATTCTCCCA[A/G]AGAAGCAATTGTTGCACTAATCAGGAACAAAATTGCA
4357_2420	C/A	TTGAACCTTCATCCCACCCTCGTCGAGGGACA[C/A]TGTGAACTGTTCAGTGTTCATCTCTGCTGAACACTGCCCACC
67935_5267	G/T	TTGACACTATAGGGGGGGCGAGGGGGGGGGCAAAGA[G/T]GACATCTGAACAAGGGATTGCAGTGGAGACTAAGACGAA
18796_271	A/G	TTGATGCCAGACAGTTTTTACCATAGTAT[A/G]TACCGGTAAAAAACCATCACGTGACATAAAGTTCCCAAGCCTTTG
11591_7956	T/G	TTGGAAAGGTTCACACTCACAGGCATCACAATCATCACA[T/G]CTTCACACCATCGGGTATTGGTCAGGTCTTTGCAC
35456_416	G/T	TTGGTCATCGGTGTAACAAACAGCAGAAC[G/T]TTACAACTGAGAAGTTGGCACAGTTTTCTGTCCAGATGTCCTAAC
112212_2270	C/G	TTGTTTTGCGTGTTTTCACATGTTCAATTGT[C/G]ACTGTGAAGTTAAACTTTTTTATCTGATTACAAATATTTTGTC
88820_8425	G/A	TTTAAAAATGTGAACAGAGTACCACCGCTCTCAATGGGC[G/A]AACCCATTACGAGTGGTGATATAAGACCCACCTCC
34060_5895	C/T	TTTAAGTGTAGCTGTTCATTTTGCAACGCATTTTGC[C/T]CCGGCAGAAAATTTGGCCAATTCTGGCCAATTTTGGTG
9209_9478	T/G	TTTATTGTGTTTAAAACATCATATCAAAACGTGAGTCTTGA[T/G]ATTTTTCTTCTTTATTCTACAGCATGTTTTCAA
33770_11226	G/A	TTTCAATAATTGCAGTCGCGTCCTGTGCTATTGAACGTCACAG[G/A]AACGCGAAACACCGGCTGTGGTAACATCACG
34181_9701	G/T	TTTCCATTGGTGCTAGACGTTGCTCTAGTAAAGCAAGACTC[G/T]GTCTCTGCCTCGTTGCCGACCAGCTCATATTCG
37539_6688	T/A	TTTCCTCACGCCGCAAGCATAAACGGCTCCGTTGCCT[T/A]TCCAGTGAGTGTGTGTTGGAAGGCTTTGCCGGTTGGC
51946_9821	A/C	TTTCGTAAAGTAAATCTAAGAATCTAGGAATCTAGGAAAAC[A/C]CGCCCAGCAAAACTACGAATCCAAGAACAAGCA

27928_8131	G/T	TTTCTCCCATCCGCCAGGCCCCGGTAGAGTACGCCAACG[G/T]GACCAAGTCGAGCTGCACTATCAGCTTCAGCTTCC
33314_3492	T/C	TTTGACCCGCACGCGGCCCACCACACCATCACA[T/C]CGGAGAATGACGTCACAATCCACTCCCGGAGTGGGCGGGTC
2384_8609	T/C	TTTGAGAAGCTTAATTTTGACCTTTGAACCCTGTTTGACC[T/C]GACCTTTGACATGACGCTTCCGTTTTTGAACACA
6201_7162	C/T	TTTGCAAGTGGTCTGGAAGGTTATGCAGGAATTAACAT[C/T]GATTCAACAATGTCGTAATTCTCCTTTAAATCCGCC
105895_9265	C/T	TTTGGTAGGCATATAAATGAATATTGGTTTCCACGAGTGT[C/T]AGAAATAAAGATATCCTTGCTCTGGTGTTTCCGT
22456_9615	C/G	TTTTAACTTACACTCAATAGGTACTTGCCGGACGTTAGAAA[C/G]GTTCTACAGAGAAAGCGGCATATTTTGCGTAAC
77007_8379	G/A	TTTTATTTCATCACAAATGTGTGCCAATGTTTCTCACTG[G/A]GCTCGGCATGTCTAATTTGCTGGCTGACTATATAA
146654_7027	T/C	TTTTCCAATGACACTGAGGACACTGATAGGCCTATAG[T/C]TACATGGCTGCGCTTTGTCTCCTGACTTAAAGATCGG
13879_207	T/C	TTTTGCAAGTGTAATGCTTCCACAATGTT[T/C]CTGAACACACAGTTACAATAATTTTGAGTCAACATGCTGATATCG
323904_3876	T/C	TTTTGCTTACAGTTTCGAAACGTAAGGAGCGAA[T/C]CGTGACTACCGTGGTCAGACAAGGCAAAAACAGGATAATTC
1573_35	A/C	TTTTGTCGATTGGCCAAATACGAATGCAA[A/C]AAGGTGTAGTAGCGGCCACAGTAATGGAGAGCGTCCAGGAGCGCT
24763_328	A/C	TTTTTAATATATGCACTGCAGAGTGCGGC[A/C]AACGTCGTGTTGTCTGTTACCTACTGACGTTGAAATTCGACATCT
75730_4484	G/A	TTTTTGCCACTTAGTGGCAAAAATTAGCATGAAT[G/A]AAATTTTATGAAGTTTTTGCAACTTCATGCAAATTTTATC
